# Polyaminoazide mixtures for the synthesis of pH-responsive calixarene nanosponges

**DOI:** 10.3762/bjoc.15.59

**Published:** 2019-03-12

**Authors:** Antonella Di Vincenzo, Antonio Palumbo Piccionello, Alberto Spinella, Delia Chillura Martino, Marco Russo, Paolo Lo Meo

**Affiliations:** 1 Dipartimento di Sicenze e Tecnologie Biologiche, Chimiche e Farmaceutiche (STEBICEF), University of Palermo – V.le delle Scienze, Ed. 17, 90128 Palermo, Italy; 2 ATeNCenter, University of Palermo – V.le delle Scienze, Ed. 18, 90128 Palermo, Italy; 3 Istituto per lo Studio dei Materiali Nanostrutturati (ISMN), CNR Palermo – Via Ugo La Malfa 153, 90146 Palermo, Italy

**Keywords:** calixarenes, nanosponges, pH-responsive materials, pollutants, polyaminoazides

## Abstract

Two mixtures of polyaminoazides were synthesized by a nucleophilic displacement strategy providing no separation of the components. The mixtures were adequately characterized by means of combined HR-ESIMS, FTIR and NMR techniques and, despite their complexity, they were successfully used to accomplish the subsequent preparation of pH-sensitive calixarene hyper-reticulated nanosponge materials. The desired responsivity to pH variations of the nanosponges obtained was verified by means of absorption tests on a set of organic pollutant model molecules.

## Introduction

The ability to provide a response to external stimuli is a highly desirable property for tailored materials designed to accomplish particular functions, such as targeted drug delivery systems, as accounted for by the present burst in literature reports [1–6]. In this context, we have been recently interested in the synthesis of nanosponge materials with tunable properties. Nanosponges (NSs) [7–14] are hyper-cross-linked polymers or copolymers obtained by reacting supramolecular host species as the monomer units with suitable reticulating agents. Of course, the structural, physical and chemical features of the obtained material critically depend on the host monomer and the linker used, on their molar ratio and, in the case of copolymers, on the molar ratio between the parent comonomers. Therefore, by a proper choice of the components, tunability in the properties of the materials obtained can be achieved. In addition, by subjecting the same materials to chemical post-modification, it is possible to introduce further groups able to provide responsivity to external stimuli. We have recently succeeded in obtaining NSs based on polyamino-cyclodextrins (ACDNs) [15] as well as mixed cyclodextrin–calixarene copolymers (CyCaNSs) [16–18], which showed pH-dependent sequestration abilities towards different model organic substrates. The latter NSs were easily synthesized by means of a CuAAC-type reaction [19–21] between a heptakisazido-β-cyclodextrin and a tetrakis(propargyloxy)calix[4]arene derivative. More recently, we also reported the synthesis of entirely synthetic calixarene nanosponges (CaNSs) [22], which can be prepared by means of the same CuAAC approach, by reacting a tetrakis(propargyloxy)calix[4]arene with a diazidoalkane. Of course, the reaction results in the formation of bis(1,2,3-triazol-1-yl)alkylidene chains as the linkers between the calixarene monomer units. Even these materials were proven effective in sequestrating organic model pollutants, such as *p*-nitroaniline derivatives or dyes, from aqueous solution. However, their synthesis required much longer times in comparison with CyCaNSs. This fact was explained assuming that a diazidoalkane is a less effective reticulating agent than a heptakisazido-β-cyclodextrin, simply because of the lesser number of reactive azide groups present. Hence, we reasoned that the polymer network formation process could have been improved by using a polyazide as the reticulating agent, and that the introduction of ionizable functionalities in structure of the latter one could be a way to provide a sensitivity to pH condition in the resulting NS material.

**Scheme 1:**
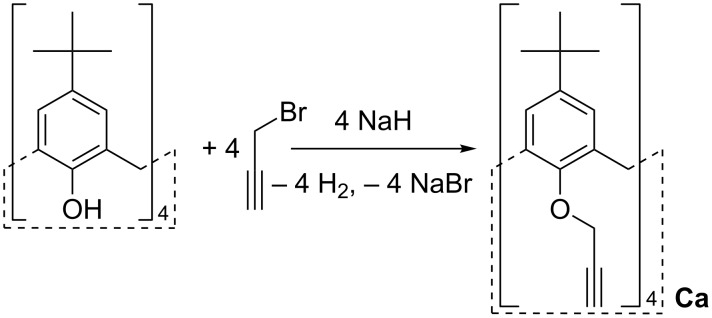
Synthesis of the propargyloxy calixarene **Ca**.

In the present work we describe the synthesis and characterization (HR-ESIMS, FTIR, NMR) of polyaminoazide mixtures, which were in turn used for the preparation of CaNSs materials with pH-tunable properties, by reaction with the (5,11,17,23-tetra(*tert*-butyl))-(25,26,27,28-tetrakis(propargyloxy)calix[4]arene (**Ca**) under the CuAAC reaction conditions. In turn, the synthon **Ca** is obtained by reacting the parent (5,11,17,23-tetra(*tert*-butyl))-(25,26,27,28-tetrahydroxy)calix[4]arene with an excess of propargyl bromide (see Scheme 1). The pH-dependent abilities of the relevant calixarene-based polymeric materials obtained were finally tested towards some suitable organic guests, suitably selected as pollutant models.

## Results and Discussion

### Synthesis and characterization of polyazidoamine mixtures

**Scheme 2:**
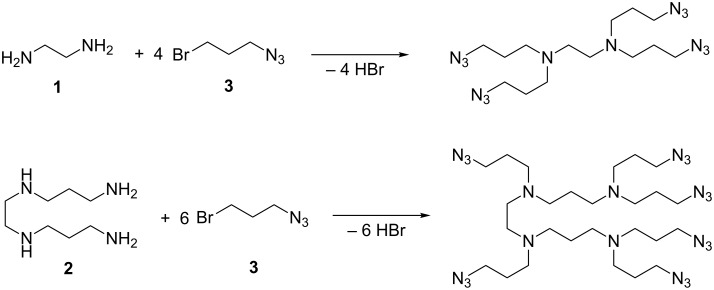
Synthesis of polyaminoazides from polyamines.

We were initially interested in the preparation of a diaminotetraazide and a tetraaminohexaazide by reaction between either 1,2-diaminoethane (**1**) or *N,N’*-bis(3-aminopropyl)-1,2-diaminoethane (**2**) and 3-azido-1-bromopropane (**3**, Scheme 2)

**Scheme 3:**
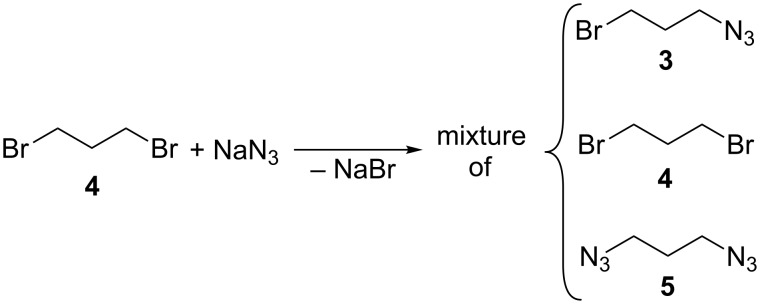
Reaction of 1,3-dibromopropane (**4**) with sodium azide.

The bromoazide **3**, in turn, can be obtained by reacting 1,3-dibromopropane (**4**) with one equivalent of sodium azide. However, even if the reaction is performed under stoichiometric conditions, it has been found that the desired product cannot be recovered (by chromatography) in yields larger than 50% [23–25]. Indeed, we verified that the reaction affords mixtures containing **3** together with the relevant diazide **5** and the unreacted starting dibromide **4** (Scheme 3), in 46%, 27% and 27% yields, respectively (estimated by ^1^H NMR), which is in reasonable agreement with the 2:1:1 ratio expected on the grounds of numerical simulations.

The separation of such a mixture would be unsuitable in view of a possible large-scale synthesis and application of the relevant nanosponge polymeric materials, because it should involve undesirable waste of time and materials. However, considering that NSs possess anyway a highly disordered structure, we reasoned that the use of a pure product as the reticulating agent would not have been a stringent requirement. So, we decided to react the aforementioned polyamines **1** and **2**, with the crude mixture described in Scheme 3. After having isolated and characterized the relevant product mixtures, indicated hereinafter as mixture **I** and **II**, respectively, the latter ones were employed as the reticulating agents for the CuAAC reaction with the aforementioned propargyloxy-calixarene **Ca**, in order to obtain two different materials, indicated as **CaNS-I** and **CaNS-II**, respectively.

Owing to their complex nature, mixtures **I** and **II** were first characterized by means of HR-ESIMS techniques. High-resolution mass spectrometry, in fact, enables to unambiguously identify the molecular formula of each compound present in the mixtures, and to determine their relative amount as well. Hence, keeping into account the structures of the precursor species, by means of a simple combinatorial analysis it is possible to show how each component is indeed the product of simple nucleophilic displacement processes. The HR-ESIMS spectra of both mixtures appear very complicated, of course; therefore, full lists of the signals present, together with the relevant abundances and possible corresponding structures are reported in Supporting Information File 1, Tables S1–S4. Analyzing for instance the mass spectra of mixture **I**, one can immediately notice the presence of signals at *m*/*z* 310.2255 and 393.2625 u, which can be attributed to the expected tri- and tetraazide derived from the reaction between diamine **1** and three or four units of **3**, respectively. The signals at *m*/*z* 267.2055 and 350.2534 u can be justified admitting the formation of a diazepane ring unit by reaction of **1** with dibromide **4**, which then reacts with two or three units of **3**.

**Scheme 4:**
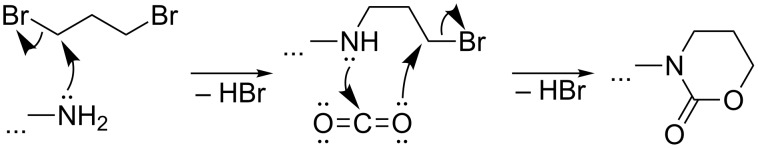
Formation of the 1,3-oxazinan-2-one ring.

The reaction of two units of diamine **1** with one unit of **4** affords a linear tetraamine, which then reacts with up to five units of **3** affording the products at *m*/*z* 410.3280, 493.3688, 576.4216 u, respectively. On the other hand, the same tetraamine may react with further dibromide units, affording other cyclic derivatives able to react with **3**. A few products can be even observed, obtained from a possible hexaamine deriving in turn by the reaction of three units of **4** with two (or even more) dibromide units. Moreover, it is interesting to notice the presence in the mixture of dioxygenated products, the formation of which might be justified admitting the reaction of a primary amine group with a dibromide **4** unit and a CO_2_ molecule (likely absorbed from atmosphere) to afford a 1,3-oxazinan-2-one ring according to Scheme 4. The CO_2_ is very likely to derive from the decomposition of the K_2_CO_3_ used as base in the reaction. In fact, we verified that the formation of similar byproducts occurs even if the reaction is carried out in a closed vessel under inert (Ar) atmosphere.

**Scheme 5:**
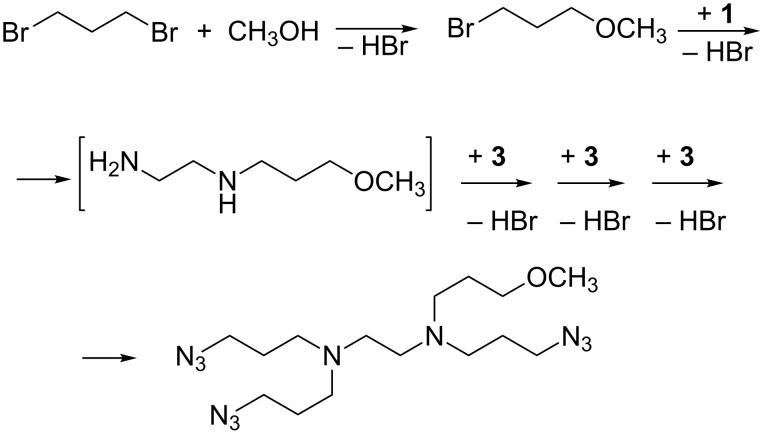
Formation of the product at *m*/*z* 382.2765 u.

**Scheme 6:**
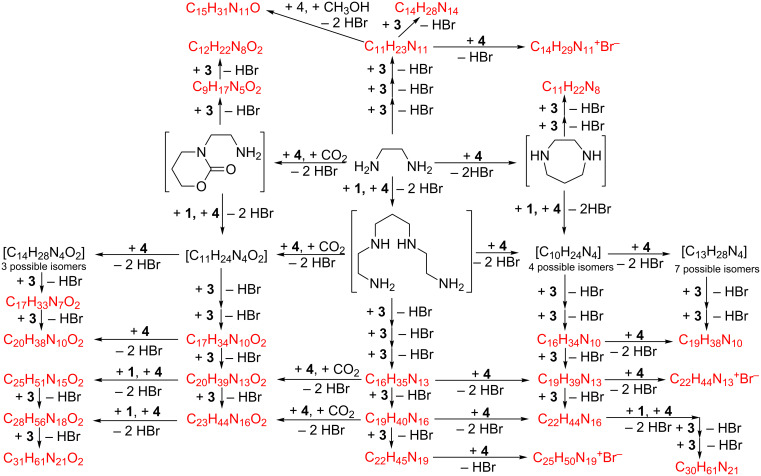
Formation of the components of mixture **I**.

Finally, the presence of a signal at *m*/*z* 382.2765 u, corresponding to the molecular formula C_15_H_31_N_11_O·H^+^, can be explained with the reaction of **1** with 1-bromo-3-methoxypropane, formed in situ by partial solvolysis of **4** (Scheme 5). A comprehensive reaction chart for mixture **I** is provided in Scheme 6, whereas the analogous chart for mixture **II** is reported in Supporting Information File 1, Scheme S1.

All the signals present in the spectra of mixture **II** can be easily explained by a suitable combination of the same aforementioned elementary processes. Up to 40 different products can be identified in detectable amounts, containing up to eight azide groups, which are obtained by either the parent tetraamine **2** or by an octaamine, derived in turn by reaction of two units of **2** with at least one unit of dibromide **4**. As a last remark, it is worth mentioning here that most of the products detected in both mixtures can indeed exist as different indistinguishable isomers (even more than 20 in some cases).

**Figure 1:**
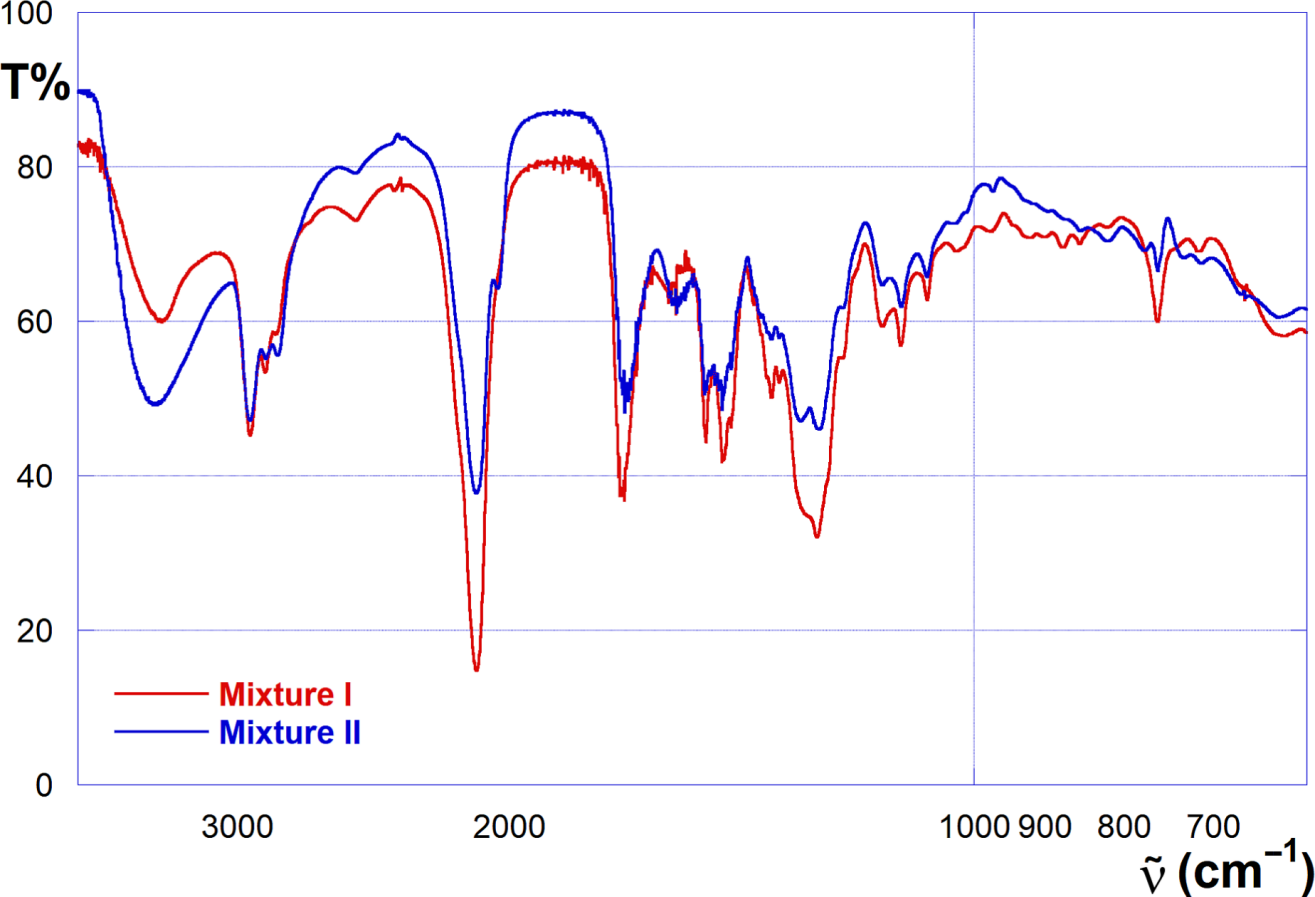
FTIR spectra (liquid) of mixture **I** (red) and mixture **II** (blue).

In order to support the interpretation of HR-ESIMS spectra, further characterization of the mixtures was achieved by means of FTIR and NMR spectroscopy. In both the FTIR spectra of mixtures **I** and **II** (Figure 1) the main feature is constituted by the presence of the characteristic -N_3_ stretching band at 2103 cm^−1^. Interestingly, spectra show also a band at ca. 1688 cm^−1^, in a position consistent with the presence of a urethane carbonyl group. The latter finding provides a confirmation of the formation of oxazinanone derivatives shown in Scheme 4.

On passing to NMR characterization (the complete collection of spectra is reported in Supporting Information File 1), the apparently very complex ^1^H NMR spectrum of mixture **I** (Supporting Information File 1, Figure S1) is characterized by the presence of various sets of signals, which are indeed easily recognizable, namely: i) a cluster of quintuplets in the range of 1.60–2.00 ppm, which can be attributed to the central -CH_2_- groups of propylene chains; ii) a cluster of signals, mainly triplets, in the range of 2.40–3.10 ppm, which can be attributed to methylene groups linked to amine N atoms; iii) a cluster of multiplets in the range of 3.20–3.50 ppm, which can be attributed to methylene groups linked to -N_3_ groups; iv) a set of three multiplets in the range of 4.08–4.22 ppm, in a position typical for methylene groups linked to oxygen atoms. These characteristics are mirrored in the relevant ^13^C NMR spectrum (Supporting Information File 1, Figure S2) by the presence of various clusters of signals in the ranges: i) 21.0–28.0 ppm (central -CH_2_- groups of propylene chains); ii) 45.0–48.5 ppm and 50.0–54.0 ppm (-CH_2_- groups bound to N atoms); iii) 66.2–66.4 ppm (-CH_2_- groups bound to O atoms). The spectrum shows also a tiny signal at 154.4 ppm that confirms the presence of the urethane carbonyl group. All these attributions were confirmed by analysis of COSY and HMQC 2D spectra (Supporting Information File 1, Figures S3 and S4). The NMR spectra of mixture **II** (see Supporting Information File 1, Figures S5–S8) show similar features as those of **I**; however, because of the greater complexity of the mixture, and of the structures of the compounds present in it as well, the spectra in general show a much worse resolution of the signals. As a final remark, it is worth mentioning here that integration analysis of the signals in the ^1^H NMR spectra of our mixtures cannot be considered enough unreliable, unfortunately, because of both the complexity of the partly overlapped signals pattern, and the concomitant overlap with the signals relevant to the solvent (DMSO and residual water).

### Synthesis and characterization of the CaNS nanosponges

**Figure 2:**
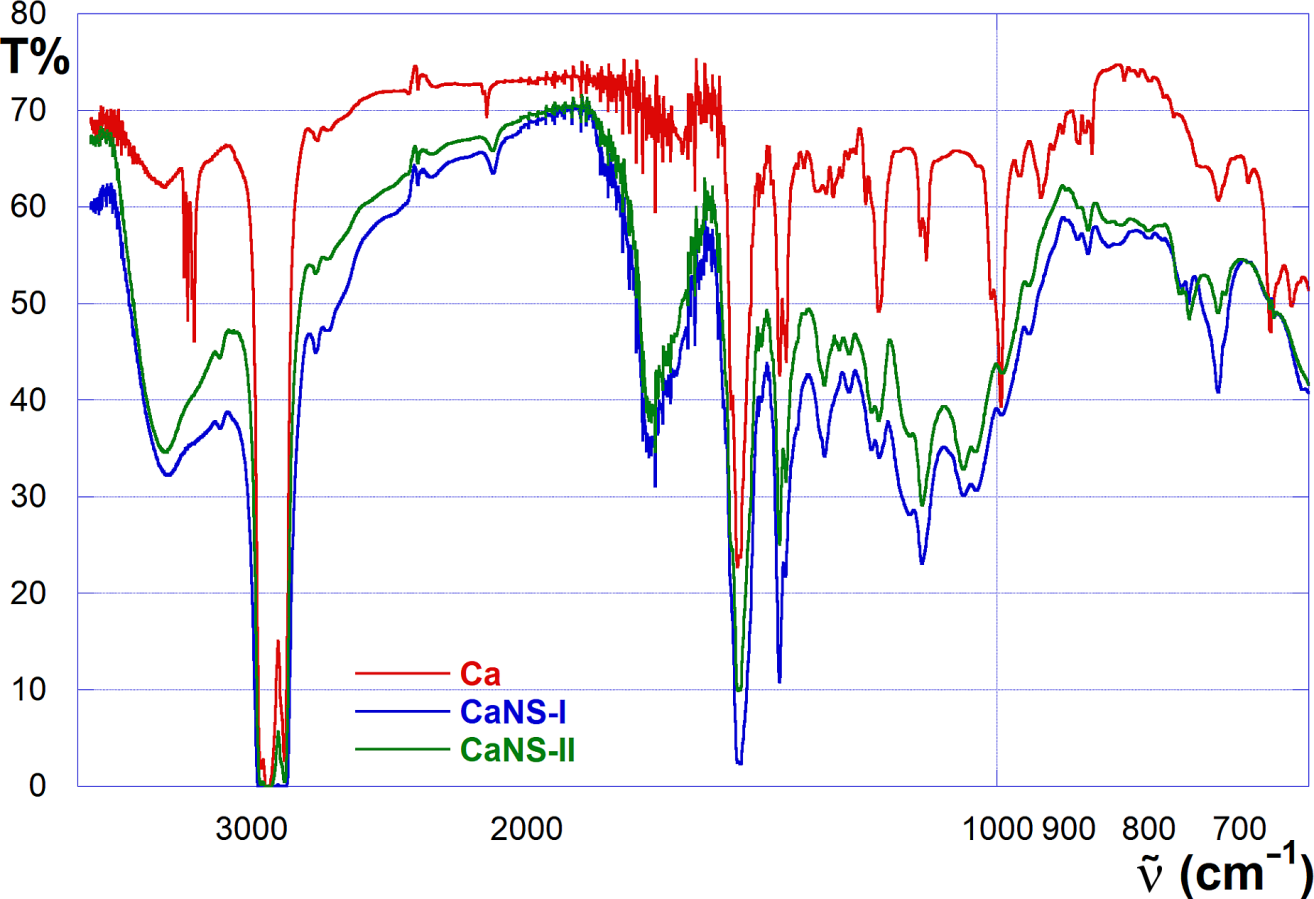
FTIR spectra (nujol) of **Ca** (red), **CaNS-I** (blue) and **CaNS-II** (green).

Having obtained the polyaminoazide mixures **I** and **II**, these were reacted with the propargyloxycalixarene **Ca** by means of the CuAAC reaction, to obtain the desired nanosponge materials **CaNS-I** and **CaNS-II**. Noticeably, by adopting the same reaction conditions used for the synthesis of CaNSs reported in our previous work [22], we observed a reduction of the reaction time needed down to 70 h. The latter finding confirms the idea that increasing the number of azide groups on the structure of the reticulating agent enhances its efficacy. Once again, combined FTIR and solid state CP-MAS ^13^C NMR confirmed the accomplishment of the reticulation reaction and the formation of the polymeric network. In particular, the IR spectrum (Figure 2) shows the disappearance of the signals in the range of 3310–3260 cm^−1^ and of the intense signal at 2103 cm^−1^, attributable to the C_sp_–H stretching of **Ca** and to the azide stretching of the reticulating agent, respectively. On the other hand, a tiny signal at 3140 cm^−1^ appears, which can be attributed to the C_sp2_–H stretching typical of the newly formed 1,2,3-triazole ring. The occurrence of the triazole ring is also accounted for by tiny signals at 1244, 1204, 1050 and 1031 cm^−1^, which can be attributed to neither the calixarene scaffold nor the polyaminoazide, whereas fingerprint signals at 1377, 1364, 1189 and 994 cm^−1^ can be attributed to the calixarene, and the signals at 1289 and 1116 cm^−1^ can be recognized as due to the linker groups

**Figure 3:**
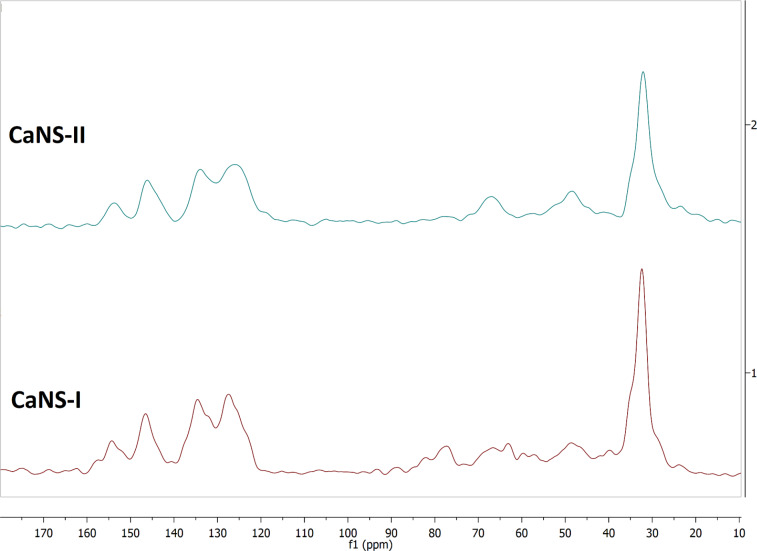
CP-MAS NMR spectra of CaNSs.

In the solid state ^13^C NMR spectra (Figure 3) it is possible to recognize the peculiar cluster of four signals in the C-aromatic region, centered at ca. 127, 134, 146 and 154 ppm, due to the aromatic C atoms of the calixarene and triazole units. On the other hand, the aliphatic region shows a main signal centered at ca. 32 ppm, the deconvolution analysis of which reveals constituted by the superimposition of at least three different signals, accounting for the *tert*-butyl groups and the methylene bridges of the calixarene units, together with the central methylene groups of the -(CH_2_)_3_- chains present in the polyaminoazide mixtures. The aliphatic region shows also other minor signals, accounting for the other C atoms present in the structure, in particular at ca. 48 and 65 ppm, which can be attributed, respectively, to C atoms bound to N or O atoms. Finally, a further signal at ca. 78 ppm may be attributed to the presence of unreacted alkyne groups.

### Sequestration abilities of the CaNS nanosponges

**Figure 4:**
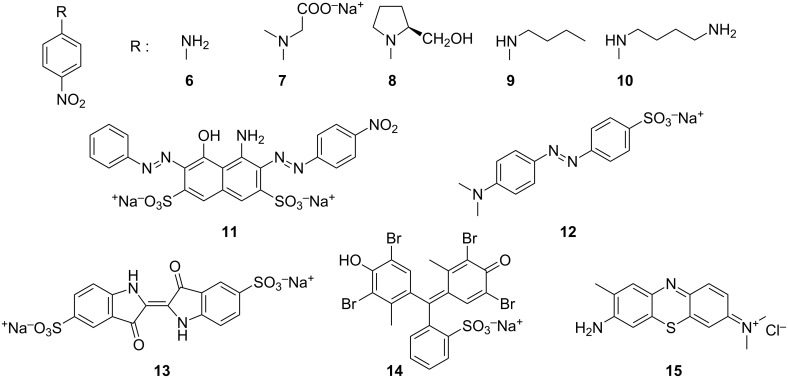
Structures of guests **6**–**15**.

The possible pH-responsive sequestration abilities of materials **CaNS-I** and **CaNS-II** were verified by means of sequestration tests on a set of suitable guest, namely *p*-nitroaniline derivatives **6**–**10** and dyes **11**–**15** (Figure 4).

These molecules were chosen as representative of different structural classes of possible guests, and show significant variations in their molecular properties such as volume, steric hindrance, hydrophobic character or charge status as a function of pH. Indeed, the same guests were already used to test the binding properties of CaNSs examined in our previous work [22], because they have been considered good models of organic pollutants [26–30]; moreover, they have been largely used as probes to investigate the microscopic interactions involved in the binding equilibria with other classes of supramolecular hosts such as calixresorcinarenes and cyclodextrins [31–38]. The results obtained (percent of guest absorbed under the operational conditions chosen, see Experimental) are summarized in Table 1.

**Table 1:** Percent of absorbed guest in the sequestration tests.^a^

guest	pH	**CaNS-I**	**CaNS-II**	guest	pH	**CaNS-I**	**CaNS-II**

**6**	4.4	21	30	**11**	4.4	>97	>97
	6.7	13	27		6.7	>97	94
	10.7	25	39	**12**	4.4	97	>97
**7**	4.4	83	88		6.7	94	96
	6.7	46	52	**13**	4.4	>97	>97
	10.7	36	53		6.7	>97	>97
**8**	4.4	54	66	**14**	4.4	>97	>97
	6.7	59	68		6.7	>97	>97
**9**	4.4	86	89	**15**	6.7	20	26
	6.7	92	95		10.7	33	43
**10**	6.7	33	37				
	10.7	54	58				

^a^All data are given with a ±3% indetermination.

As expected, both materials may show remarkable variations in their sequestration abilities (as accounted for by the percent of sequestered guest) on varying the pH value of the solvent medium. However, these data deserve detailed discussion, even in comparison with the results obtained in our previous work. For instance, the inclusion of neutral *p*-nitroaniline guests **6**, **8** and **9** shows only a fair dependence on pH. By contrast, inclusion of the charged guests **7** and **10** presents remarkable variations with pH, which are not mirrored by analogous non-aminated calixarene polymers examined in our previous work [22]. More in detail, inclusion of anionic **7** decreases on increasing the pH, clearly indicating that inclusion is favorably affected by the presence of positive charges on the polymer network due to protonation of amine basic sites. Consistently, the behavior of the diamine derivative **10** is reversed (inclusion decreases on decreasing the pH). A comparison between guests **9** and **10**, which differ only for the presence of the -NH_2_ tail group, is interesting. In fact, the amine group makes the guest more hydrophilic and able to be protonated; as a consequence, **10** is sequestrated much less effectively. Consistently, the behavior of dyes **11**–**15** confirms the importance of electrostatic interactions in the sequestration process. In fact, anions **11**–**14** are sequestered nearly quantitatively from solution. By contrast, cationic **15** is absorbed much less effectively and, as expected, binding is favored under the most alkaline pH conditions.

## Conclusion

In the present work we verified the feasibility of synthesizing new pH-responsive calixarene nanosponges by using two different polyaminoazide mixtures as the reticulating agent. Full characterization (HR-ESIMS, FTIR, 1D and 2D NMR) of the mixtures has been provided. It is particularly important to stress that the use of either mixture led to the formation of the desired hyper-reticulated, microscopically disordered, polymeric network in a fully satisfactory way, despite their very complex composition (at least 20 different species present in detectable amount for mixture **I**, at least 40 for mixture **II**). The relevant nanosponges **CaNS-I** and **CaNS-II** were similarly subjected to spectroscopic characterization (FTIR, solid-state CP-MAS NMR), whereas their pH-dependent absorption abilities were verified towards typical probe guests such as *p*-nitroaniline derivatives and commercial dyes. Our results indicate an excellent affinity of our materials towards anionic guests in general, once again confirming that Coulomb interactions assume a paramount role in affecting the absorption equilibrium at a microscopic level.

Finally, it is also important outlining, in our opinion, that the possibility to introduce pH-sensitive functionalities in the highly hydrophobic nanoporous environment provided by the fully synthetic calixarene-based polymer, might open interesting perspectives for applicative purposes, in particular in the fields of organocatalysis and of hybrid organic-inorganic materials, such as in the emerging area of the nanosponge–metal nanoparticles conjugates.

## Experimental

**Materials**. All the reagents and solvents needed were used as purchased (Aldrich, Fluka) without further purification. The calixarene derivative **Ca** [39] and the non-commercial nitroaniline derivatives **7**–**10** [31,33] were prepared according to the procedures reported in literature.

**Instrumentation**. UV–vis spectra were recorded on a Beckmann Coulter DU 800 spectrophotometer, equipped with a Peltier thermostatic apparatus. FTIR spectra on powders were acquired on a Bruker VERTEX70 instrumentation. NMR spectra were recorded on a Bruker Avance III (600 MHz) spectrometer; the solid-state spectra were acquired with a 15 kHz rotating MAS probe. High resolution ESI mass spectra were acquired in positive mode on an AGILENT Technologies 6540 UHD Accurate Mass Q-TOF LC–MS apparatus (1 kV nozzle voltage, 175 V fragmentor voltage).

**Synthesis of polyaminoazide mixture I**. 1,3-Dibromopropane (**4**, 1.0 mL, 1.98 g, 9.8 mmol) and NaN_3_ (0.65 g, 10 mmol) were dissolved in methanol (10 mL), and the mixture was stirred at 45 °C for 24 h. Then 1,2-diaminoethane (**1**, 167 μL, 150 mg, 2.5 mmol) and K_2_CO_3_ (1.38 g, 10 mmol) were added and the mixture was stirred at rt for 72 h. The reaction crude was poured into water (50 mL) and filtered. The solution is extracted thrice with diethyl ether (ca. 40 mL each), whereas the solid was washed with diethyl ether too. The combined organic extracts were washed with brine, dried over Na_2_SO_4_, and finally distilled in vacuo to afford the product, which was not subjected to further purifications before use. Yield 0.80 g.

**Synthesis of polyaminoazide mixture II**. 1,3-Dibromopropane (**4**, 1.5 mL, 2.97 g, 14.7 mmol) and NaN_3_ (0.975 g, 15 mmol) were dissolved in methanol (12 mL), and the mixture was stirred at 45 °C for 24 h. Then *N,N’*-bis(3-aminopropyl)-1,2-diaminoethane (**2**, 460 μL, 437 mg, 2.5 mmol) and K_2_CO_3_ (2.07 g, 15 mmol) were added and the mixture was stirred at rt for 72 h. The reaction crude is poured into water (50 mL) and filtered. The solution was extracted thrice with diethyl ether (ca. 40 mL each), whereas the solid was washed with diethyl ether too. The combined organic extracts were washed with brine, dried on Na_2_SO_4_, and finally distilled in vacuo to afford the product, which was not subjected to further purifications before use. Yield 1.62 g.

**Synthesis of nanosponges CaNS-I and CaNS-II**. These syntheses were accomplished in an analogous way as described in our previous work [22]. The starting calixarene **Ca** (200.0 mg, 0.25 mmol), CuSO_4_·5H_2_O (40.0 mg, 0.16 mmol) and sodium ascorbate (62.0 mg, 0.31 mmol) were mixed in a screw-cap dark vial; then the proper polyaminoazide mixture was added (either mixture **I** or mixture **II**, 200 mg), and the reactants were finally dissolved with DMSO (2 mL). The system was kept under magnetic stirring at 65 °C for 70 h. The reaction crude was then poured into water (60 mL), sonicated for a few minutes and centrifuged at 5500 rpm for 15 min. The solid was decanted, suspended in water (50 mL) sonicated for 10 min and centrifuged again. The solid was then subjected to two further suspension–sonication–centrifugation washing cycles, with methanol (50 mL) and diethyl ether (50 mL). The product was finally recovered by filtration in vacuo, grinded in a mortar, passed through a 150 μm sieve and dried in vacuo over P_2_O_5_ at 50 °C. Yield: 320 mg for **CaNS-I**, 334 mg for **CaNS-II**.

**Sequestration tests**. As described in our previous papers [17,22], stock 50 μM solutions of the guests were prepared in aqueous buffers at the required pH value. Samples were prepared by mixing 2 mL of guest solution with a carefully weighed amount (4.00 ± 0.05 mg) of material. The samples were shaken at room temperature for 90 min, and then centrifuged for 15 min at 5500 rpm. The supernatant liquor was carefully pipetted after centrifugation, and then the percent amount of guest left in solution at equilibrium was estimated by means of UV–vis spectrophotometry, by simply comparing the absorbances of the starting and final solutions (a typical example is provided in Supporting Information File 1, Figure S9); hence, the percent absorbed is trivially obtained by difference.

## Supporting Information

File 1 Possible structures of the components present in the mixtures, formation of compounds, HRMS and NMR spectra.
