# Supplementary material for: Polyaminoazide mixtures for the synthesis of pH-responsive calixarene nanosponges
Source: Beilstein J Org Chem. 2019 Mar 12;15:633–41. doi: 10.3762/bjoc.15.59 (PMC6423589; doi:10.3762/bjoc.15.59)
Supplement: File 1 — Possible structures of the components present in the mixtures, formation of compounds, HRMS and NMR spectra. [file Beilstein_J_Org_Chem-15-633-s001.pdf]

BEILSTEIN JOURNAL OF ORGANIC CHEMISTRY

Supporting Information

for

Polyaminoazide mixtures for the synthesis of pH-responsive calixarene nanosponges

Antonella Di Vincenzo, Antonio Palumbo Piccionello, Alberto Spinella, Delia Chillura Martino, Marco Russo and Paolo Lo Meo

Beilstein J. Org. Chem. **2019,** *15,* 633–641. doi:10.3762/bjoc.15.59

Possible structures of the components present in the mixtures, formation of compounds, HRMS and NMR spectra

License and Terms: This is a supporting information file under the terms of the Creative Commons Attribution License (http://creativecommons.org/licenses/by/4.0). Please note that the reuse, redistribution and reproduction in particular requires that the authors and source are credited. The license is subject to the *Beilstein Journal of Organic Chemistry* terms and conditions: (https://www.beilstein-journals.org/bjoc)

Contents

Table S1: Signals in the HR-ESIMS spectra of mixture **I**.

Table S2: Possible structures of the components present in mixture **I**.

Table S3: Signals in the HR-ESIMS-spectra of mixture **II**.

Table S4: Possible structures of the components present in mixture **II**.

Scheme S1: Formation of the components of mixture **II**.

Figure S1: ^{1}H NMR spectrum (DMSO, 600 MHz) of mixture **I**.

Figure S2: ^{13}C NMR spectrum (DMSO, 600 MHz) of mixture **I**.

Figure S3: COSY NMR spectrum (DMSO, 600 MHz) of mixture **I**.

Figure S4: HMQC NMR spectrum (DMSO, 600 MHz) of mixture **I**.

Figure S5: ^{1}H NMR spectrum (DMSO, 600 MHz) of mixture **II**.

Figure S6: ^{13}C NMR spectrum (DMSO, 150 MHz) of mixture **II**.

Figure S7: COSY NMR spectrum (DMSO, 600 MHz) of mixture **II**.

Figure S8: HMQC NMR spectrum (DMSO, 600 MHz) of mixture **II**.

Figure S9: UV–vis spectrum of guest **7** before (red) and after (blue) equilibration with material CaNS-II.

Table S1: Signals in the HR-ESIMS spectra of mixture **I**.

m/z	calculated	molecular formula	structure[a]	mole fraction %
228.1461	228.1455	$C_9H_{17}N_5O_2 \cdot H^+$	***ACD***H	1.0
267.2055	267.2040	$C_{11}H_{22}N_8 \cdot H^+$	***ABD***$_2$	1.4
310.2255	310.2211	$C_{11}H_{23}N_{11} \cdot H^+$	***AD***$_3$H	6.3
311.1988	311.1938	$C_{12}H_{22}N_8O_2 \cdot H^+$	***ACD***$_2$	5.0
350.2534	350.2529	$C_{14}H_{29}N_{11}{}^+$	***ABD***$_3{}^+$	2.9
367.3097	367.3046	$C_{16}H_{34}N_{10} \cdot H^+$	***A***$_2$***B***$_2$***D***$_2$H$_2$	3.2
368.2785	368.2768	$C_{17}H_{33}N_7O_2 \cdot H^+$	***A***$_2$***B***$_2$***CD***H	1.5
382.2765	382.2791	$C_{15}H_{31}N_{11}O \cdot H^+$	***ABD***$_3$(OCH$_3$)	1.4
393.2625	393.2694	$C_{14}H_{28}N_{14} \cdot H^+$	***AD***$_4$	9.2
407.3354	407.3354	$C_{19}H_{38}N_{10} \cdot H^+$	***A***$_2$***B***$_3$***D***$_2$	2.3
410.3280	410.3211	$C_{16}H_{35}N_{13} \cdot H^+$	***A***$_2$***BD***$_3$H$_3$	2.2
411.2982	411.2939	$C_{17}H_{34}N_{10}O_2 \cdot H^+$	***A***$_2$***BCD***$_2$H$_2$	2.0
450.3525	450.3524	$C_{19}H_{39}N_{13} \cdot H^+$	***A***$_2$***B***$_2$***D***$_3$H	4.9
451.3244	451.3252	$C_{20}H_{38}N_{10}O_2 \cdot H^+$	***A***$_2$***B***$_2$***CD***$_2$	3.0
490.3801	490.3837	$C_{22}H_{44}N_{13}{}^+$	***A***$_2$***B***$_3$***D***$_3{}^+$	3.2
493.3688	493.3695	$C_{19}H_{40}N_{16} \cdot H^+$	***A***$_2$***BD***$_4$H$_2$	2.5
494.3485	494.3422	$C_{20}H_{39}N_{13}O_2 \cdot H^+$	***A***$_2$***BCD***$_3$H	2.9
533.4010	533.4008	$C_{22}H_{44}N_{16} \cdot H^+$	***A***$_2$***B***$_2$***D***$_4$	5.3
576.4216	576.4178	$C_{22}H_{45}N_{19} \cdot H^+$	***A***$_2$***BD***$_5$H	1.7
577.3874	577.3906	$C_{23}H_{44}N_{16}O_2 \cdot H^+$	***A***$_2$***BCD***$_4$	3.3
594.4454	594.4423	$C_{25}H_{51}N_{15}O_2 \cdot H^+$	***A***$_3$***B***$_2$***CD***$_3$H$_3$	2.7
616.4429	616.4491	$C_{25}H_{50}N_{19}{}^+$	***A***$_2$***B***$_2$***D***$_5{}^+$	1.8
677.4795	677.4906	$C_{28}H_{56}N_{18}O_2 \cdot H^+$	***A***$_3$***B***$_2$***CD***$_4$H$_2$	4.2
716.5489	716.5497	$C_{30}H_{61}N_{21} \cdot H^+$	***A***$_3$***B***$_3$***D***$_5$H	6.6
760.5557	760.5390	$C_{31}H_{61}N_{21}O_2 \cdot H^+$	***A***$_3$***B***$_2$***CD***$_5$H	7.7

[a] ***A*** = >N(CH$_2$)$_2$N< ; ***B*** = -(CH$_2$)$_3$- ; ***C*** = -(CH$_2$)$_3$-O-CO- ; ***D*** = -(CH$_2$)$_3$N$_3$

Table S2: Possible structures of the components present in mixture **I**.

m/z	molecular formula	Possible structure[a] (number of possible isomers)
228.1461	$C_9H_{17}N_5O_2{\cdot}H^+$	***ACD***H 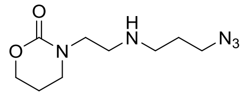
267.2055	$C_{11}H_{22}N_8{\cdot}H^+$	***ABD***$_2$ 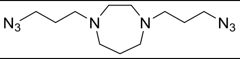
310.2255	$C_{11}H_{23}N_{11}{\cdot}H^+$	***AD***$_3$H 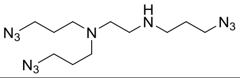
311.1988	$C_{12}H_{22}N_8O_2{\cdot}H^+$	***ACD***$_2$ 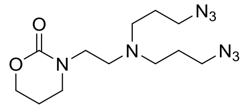
350.2534	$C_{14}H_{29}N_{11}^+$	***ABD***$_3^+$ 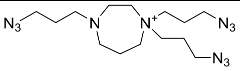
367.3097	$C_{16}H_{34}N_{10}{\cdot}H^+$	***A***$_2$***B***$_2$***D***$_2$H$_2$ 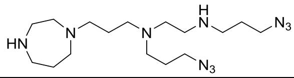 (13)
368.2785	$C_{17}H_{33}N_7O_2{\cdot}H^+$	***A***$_2$***B***$_2$***CD***H 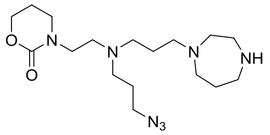 (3)
382.2765	$C_{15}H_{31}N_{11}O{\cdot}H^+$	***ABD***$_3$(OCH$_3$) 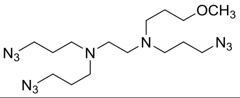
393.2625	$C_{14}H_{28}N_{14}{\cdot}H^+$	***AD***$_4$ 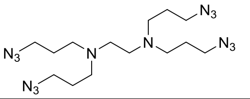
407.3354	$C_{19}H_{38}N_{10}{\cdot}H^+$	***A***$_2$***B***$_3$***D***$_2$ 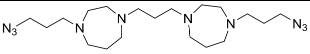 (3)
410.3280	$C_{16}H_{35}N_{13}{\cdot}H^+$	***A***$_2$***BD***$_3$H$_3$ 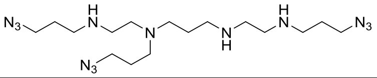 (5)
411.2982	$C_{17}H_{34}N_{10}O_2{\cdot}H^+$	***A***$_2$***BCD***$_2$H$_2$ 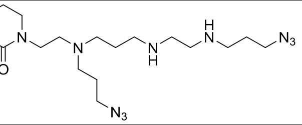 (4)
450.3525	$C_{19}H_{39}N_{13}{\cdot}H^+$	***A***$_2$***B***$_2$***D***$_3$H 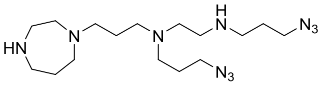 (8)
451.3244	$C_{20}H_{38}N_{10}O_2{\cdot}H^+$	***A***$_2$***B***$_2$***CD***$_2$ 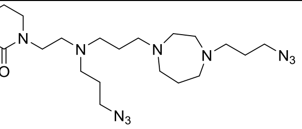 (3)
490.3801	$C_{22}H_{44}N_{13}^+$	***A***$_2$***B***$_3$***D***$_3^+$ 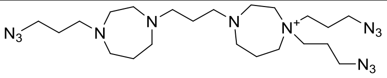 (5)
493.3688	$C_{19}H_{40}N_{16}{\cdot}H^+$	***A***$_2$***BD***$_4$H$_2$ 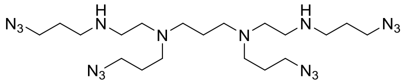 (5)
494.3485	$C_{20}H_{39}N_{13}O_2{\cdot}H^+$	***A***$_2$***BCD***$_3$H 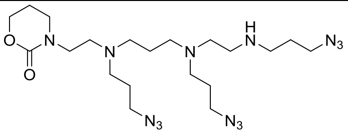 (3)
533.4010	$C_{22}H_{44}N_{16}{\cdot}H^+$	***A***$_2$***B***$_2$***D***$_4$ 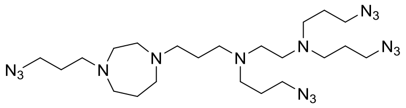 (3)

576.4216	$C_{22}H_{45}N_{19}\cdot H^+$	***A***$_2$***BD***$_5$H	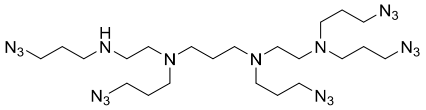	(2)
577.3874	$C_{23}H_{44}N_{16}O_2\cdot H^+$	***A***$_2$***BCD***$_4$	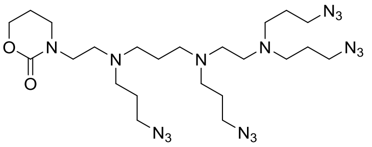	
594.4454	$C_{25}H_{50}N_{15}O_2\cdot H^+$	***A***$_3$***B***$_2$***CD***$_3$H$_3$	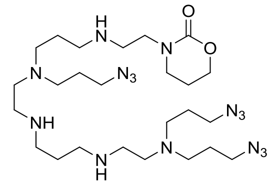	(>20)
616.4429	$C_{25}H_{50}N_{19}^+$	***A***$_2$***B***$_2$***D***$_5$$^+$	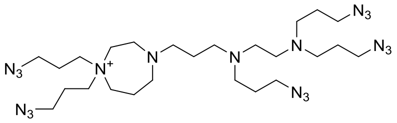	
677.4795	$C_{28}H_{56}N_{18}O_2\cdot H^+$	***A***$_3$***B***$_2$***CD***$_4$H$_2$	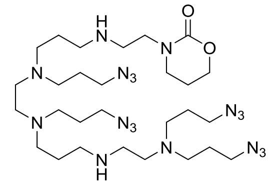	(>20)
716.5489	$C_{30}H_{61}N_{21}\cdot H^+$	***A***$_3$***B***$_3$***D***$_5$H	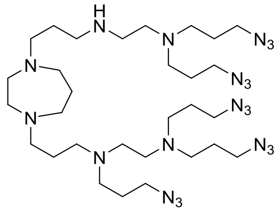	(>20)
760.5557	$C_{31}H_{61}N_{21}O_2\cdot H^+$	***A***$_3$***B***$_2$***CD***$_5$H	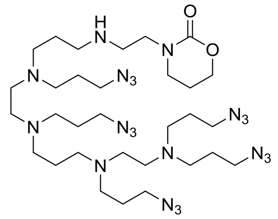	(>20)

[a] ***A*** = >N(CH$_2$)$_2$N< ; ***B*** = -(CH$_2$)$_3$- ; ***C*** = -(CH$_2$)$_3$-O-CO- ; ***D*** = -(CH$_2$)$_3$N$_3$

Table S3: Signals in the HR-ESIMS spectra of mixture **II**.

m/z	calculated	molecular formula	structure[a]	mole fraction %
342.2615	342.2612	$C_{15}H_{31}N_7O_2\cdot H^+$	$\boldsymbol{ACD}\mathrm{H}_3$	0.8
343.2344	343.2340	$C_{16}H_{30}N_4O_4\cdot H^+$	$\boldsymbol{AC}_2\mathrm{H}_2$	0.8
381.3187	381.3197	$C_{17}H_{36}N_{10}\cdot H^+$	$\boldsymbol{ABD}_2\mathrm{H}_2$	0.9
382.2939	382.2925	$C_{18}H_{35}N_7O_2\cdot H^+$	$\boldsymbol{ABCD}\mathrm{H}$	2.3
383.2657	383.2635	$C_{19}H_{34}N_4O_4\cdot H^+$	$\boldsymbol{ABC}_2$	1.7
424.3345	424.3368	$C_{17}H_{37}N_{13}\cdot H^+$	$\boldsymbol{AD}_3\mathrm{H}_3$	1.1
425.3093	425.3095	$C_{18}H_{36}N_{10}O_2\cdot H^+$	$\boldsymbol{ACD}_2\mathrm{H}_2$	1.7
426.2824	426.2823	$C_{19}H_{35}N_7O_4\cdot H^+$	$\boldsymbol{AC}_2\boldsymbol{D}\mathrm{H}$	5.2
464.3662	464.3681	$C_{20}H_{41}N_{13}\cdot H^+$	$\boldsymbol{ABD}_3\mathrm{H}$	1.7
465.3436	465.3408	$C_{21}H_{40}N_{10}O_2\cdot H^+$	$\boldsymbol{ABCD}_2$	4.6
466.3123	466.3136	$C_{22}H_{40}N_7O_4^+$	$\boldsymbol{ABC}_2\boldsymbol{D}^+$	0.6
507.3851	507.3851	$C_{20}H_{42}N_{16}\cdot H^+$	$\boldsymbol{AD}_4\mathrm{H}_2$	1.5
508.3596	508.3579	$C_{21}H_{41}N_{13}O_2\cdot H^+$	$\boldsymbol{ACD}_3\mathrm{H}$	3.1
509.3380	509.3307	$C_{22}H_{40}N_{10}O_4\cdot H^+$	$\boldsymbol{AC}_2\boldsymbol{D}_2$	9.0
547.4163	547.4164	$C_{23}H_{46}N_{16}\cdot H^+$	$\boldsymbol{ABD}_4$	2.5
548.3918	548.3892	$C_{24}H_{46}N_{13}O_2^+$	$\boldsymbol{ABCD}_3^+$	2.8
590.4328	590.4335	$C_{23}H_{47}N_{19}\cdot H^+$	$\boldsymbol{AD}_5\mathrm{H}$	1.4
591.4064	491.4062	$C_{24}H_{46}N_{16}O_2\cdot H^+$	$\boldsymbol{ACD}_4$	8.9
630.4642	630.4648	$C_{26}H_{52}N_{19}^+$	$\boldsymbol{ABD}_5^+$	1.7
673.4812	673.4818	$C_{26}H_{52}N_{22}\cdot H^+$	$\boldsymbol{AD}_6$	2.6
719.5888	719.5879	$C_{35}H_{70}N_{14}O_2\cdot H^+$	$\boldsymbol{A}_2\boldsymbol{B}_3\boldsymbol{CD}_2\mathrm{H}_2$	2.0
762.6042	762.6054	$C_{35}H_{71}N_{17}O_2\cdot H^+$	$\boldsymbol{A}_2\boldsymbol{B}_2\boldsymbol{CD}_3\mathrm{H}_3$	3.2
801.6630	801.6635	$C_{37}H_{76}N_{20}\cdot H^+$	$\boldsymbol{A}_2\boldsymbol{B}_3\boldsymbol{D}_4\mathrm{H}_2$	1.1
802.6391	802.6362	$C_{38}H_{75}N_{17}O_2\cdot H^+$	$\boldsymbol{A}_2\boldsymbol{B}_3\boldsymbol{CD}_3\mathrm{H}$	2.6
805.6225	805.6220	$C_{35}H_{72}N_{20}O_2\cdot H^+$	$\boldsymbol{A}_2\boldsymbol{BCD}_4\mathrm{H}_4$	1.5
844.6776	844.6805	$C_{37}H_{77}N_{23}\cdot H^+$	$\boldsymbol{A}_2\boldsymbol{B}_2\boldsymbol{D}_5\mathrm{H}_3$	1.2
845.6531	845.6533	$C_{38}H_{76}N_{20}O_2\cdot H^+$	$\boldsymbol{A}_2\boldsymbol{B}_2\boldsymbol{CD}_4\mathrm{H}_2$	3.2
884.7081	884.7118	$C_{40}H_{81}N_{23}\cdot H^+$	$\boldsymbol{A}_2\boldsymbol{B}_3\boldsymbol{D}_5\mathrm{H}$	1.1
885.6891	885.6846	$C_{41}H_{80}N_{20}O_2\cdot H^+$	$\boldsymbol{A}_2\boldsymbol{B}_3\boldsymbol{CD}_4$	1.1
888.6719	888.6703	$C_{38}H_{77}N_{23}O_2\cdot H^+$	$\boldsymbol{A}_2\boldsymbol{BCD}_5\mathrm{H}_3$	2.3
927.7258	927.7289	$C_{40}H_{82}N_{26}^+\cdot H^+$	$\boldsymbol{A}_2\boldsymbol{B}_2\boldsymbol{D}_6\mathrm{H}_2$	1.2
928.7016	928.7016	$C_{41}H_{81}N_{23}O_2\cdot H^+$	$\boldsymbol{A}_2\boldsymbol{B}_2\boldsymbol{CD}_5\mathrm{H}$	4.0
967.7602	967.7602	$C_{43}H_{86}N_{26}\cdot H^+$	$\boldsymbol{A}_2\boldsymbol{B}_3\boldsymbol{D}_6$	1.1
971.7187	971.7187	$C_{41}H_{82}N_{26}O_2\cdot H^+$	$\boldsymbol{A}_2\boldsymbol{BCD}_6\mathrm{H}_2$	2.6
1010.7772	1010.7772	$C_{43}H_{87}N_{29}\cdot H^+$	$\boldsymbol{A}_2\boldsymbol{B}_2\boldsymbol{D}_7\mathrm{H}$	1.4
1011.7500	1011.7500	$C_{44}H_{86}N_{26}O_2\cdot H^+$	$\boldsymbol{A}_2\boldsymbol{B}_2\boldsymbol{CD}_6$	4.0
1054.7670	1054.7670	$C_{44}H_{87}N_{29}O_2\cdot H^+$	$\boldsymbol{A}_2\boldsymbol{BCD}_7\mathrm{H}$	2.5
1093.8255	1093.8255	$C_{46}H_{92}N_{32}\cdot H^+$	$\boldsymbol{A}_2\boldsymbol{B}_2\boldsymbol{D}_8$	1.2
1094.7983	1094.7983	$C_{47}H_{92}N_{29}O_2$	$\boldsymbol{A}_2\boldsymbol{B}_2\boldsymbol{CD}_7^+$	1.4
1137.8154	1137.8154	$C_{47}H_{92}N_{32}O_2\cdot H^+$	$\boldsymbol{A}_2\boldsymbol{BCD}_8$	2.0

[a]***A*** = $>N(CH_2)_3\text{-}N(CH_2)_2N\text{-}(CH_2)_3N<$; ***B*** = $\text{-}(CH_2)_3\text{-}$; ***C*** = $\text{-}(CH_2)_3\text{-O-CO-}$; ***D*** = $\text{-}(CH_2)_3N_3$

Table S4: Possible structures of the components present in mixture **II**.[a]

m/z	molecular formula	Possible structure[b]
342.2615	$C_{15}H_{31}N_7O_2\cdot H^+$	***ACD***H_3 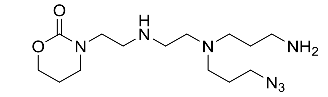
343.2344	$C_{16}H_{30}N_4O_4\cdot H^+$	***AC***$_2H_2$ 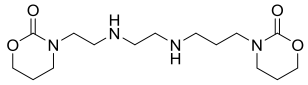
381.3187	$C_{17}H_{36}N_{10}\cdot H^+$	***ABD***$_2H_2$ 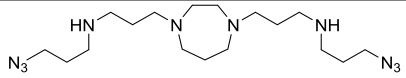
382.2939	$C_{18}H_{35}N_7O_2\cdot H^+$	***ABCD***H 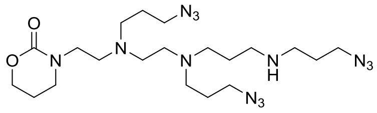
383.2657	$C_{19}H_{34}N_4O_4\cdot H^+$	***ABC***$_2$ 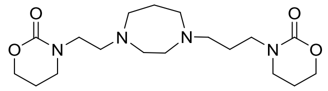
424.3345	$C_{17}H_{37}N_{13}\cdot H^+$	***AD***$_3H_3$ 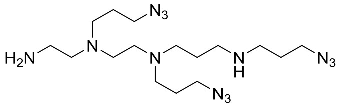
425.3093	$C_{18}H_{36}N_{10}O_2\cdot H^+$	***ACD***$_2H_2$ 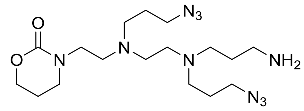
426.2824	$C_{19}H_{35}N_7O_4\cdot H^+$	***AC***$_2$***D***H 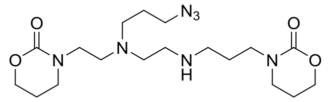
464.3662	$C_{20}H_{41}N_{13}\cdot H^+$	***ABD***$_3$H 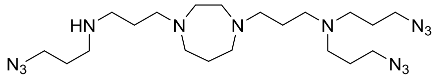
465.3436	$C_{21}H_{40}N_{10}O_2\cdot H^+$	***ABCD***$_2$ 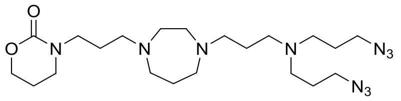
466.3123	$C_{22}H_{40}N_7O_4^+$	***ABC***$_2$***D***$^+$ 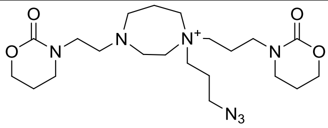
507.3851	$C_{20}H_{42}N_{16}\cdot H^+$	***AD***$_4H_2$ 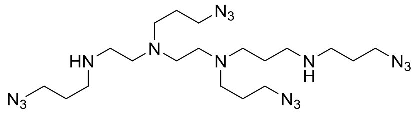
508.3596	$C_{21}H_{41}N_{13}O_2\cdot H^+$	***ACD***$_3$H 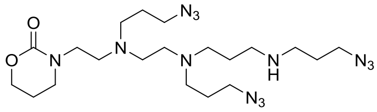
509.3380	$C_{22}H_{40}N_{10}O_4\cdot H^+$	***AC***$_2$***D***$_2$ 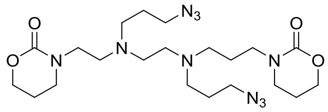
547.4163	$C_{23}H_{46}N_{16}\cdot H^+$	***ABD***$_4$ 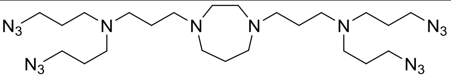

548.3918	$C_{24}H_{46}N_{13}O_2^+$	$\boldsymbol{ABCD}_3^+$	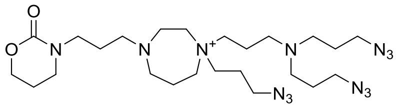
590.4328	$C_{23}H_{47}N_{19}\cdot H^+$	$\boldsymbol{AD}_5\mathrm{H}$	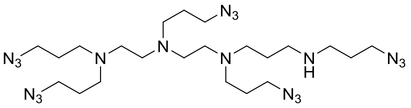
591.4064	$C_{24}H_{46}N_{16}O_2\cdot H^+$	$\boldsymbol{ACD}_4$	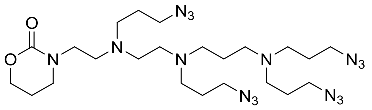
630.4642	$C_{26}H_{52}N_{19}^+$	$\boldsymbol{ABD}_5^+$	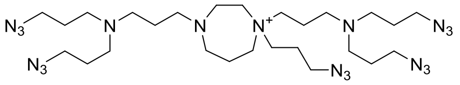
673.4812	$C_{26}H_{52}N_{22}\cdot H^+$	$\boldsymbol{AD}_6$	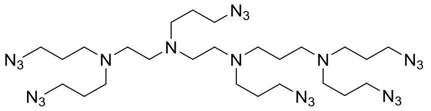
719.5888	$C_{35}H_{70}N_{14}O_2\cdot H^+$	$\boldsymbol{A}_2\boldsymbol{B}_3\boldsymbol{CD}_2\mathrm{H}_2$	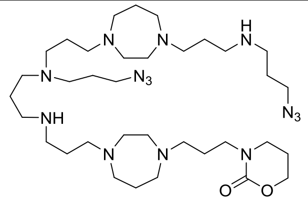
762.6042	$C_{35}H_{71}N_{17}O_2\cdot H^+$	$\boldsymbol{A}_2\boldsymbol{B}_2\boldsymbol{CD}_3\mathrm{H}_3$	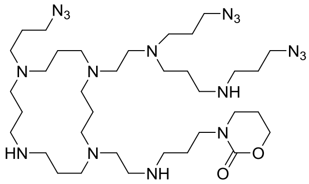
801.6630	$C_{37}H_{76}N_{20}\cdot H^+$	$\boldsymbol{A}_2\boldsymbol{B}_3\boldsymbol{D}_4\mathrm{H}_2$	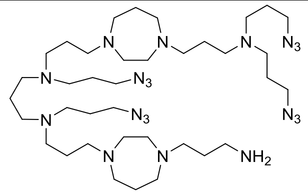
802.6391	$C_{38}H_{75}N_{17}O_2\cdot H^+$	$\boldsymbol{A}_2\boldsymbol{B}_3\boldsymbol{CD}_3\mathrm{H}$	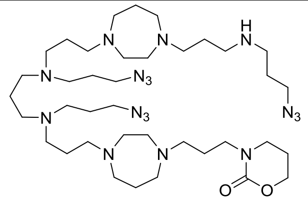
805.6225	$C_{35}H_{72}N_{20}O_2\cdot H^+$	$\boldsymbol{A}_2\boldsymbol{BCD}_4\mathrm{H}_4$	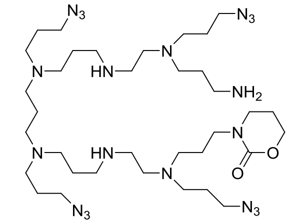
844.6776	$C_{37}H_{77}N_{23}\cdot H^+$	$\boldsymbol{A}_2\boldsymbol{B}_2\boldsymbol{D}_5\mathrm{H}_3$	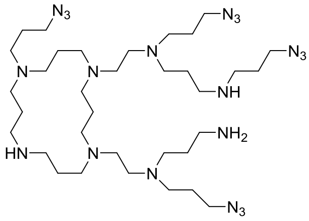

845.6531	$C_{38}H_{76}N_{20}O_2\cdot H^+$	$\boldsymbol{A}_2\boldsymbol{B}_2\boldsymbol{C}\boldsymbol{D}_4H_2$	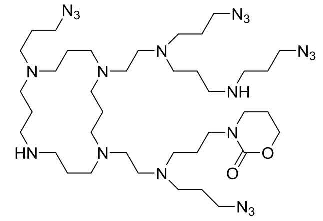
884.7081	$C_{40}H_{81}N_{23}\cdot H^+$	$\boldsymbol{A}_2\boldsymbol{B}_3\boldsymbol{D}_5H$	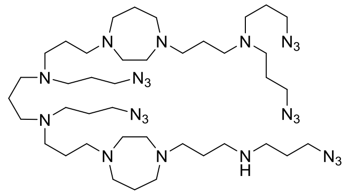
885.6891	$C_{41}H_{80}N_{20}O_2\cdot H^+$	$\boldsymbol{A}_2\boldsymbol{B}_3\boldsymbol{C}\boldsymbol{D}_4$	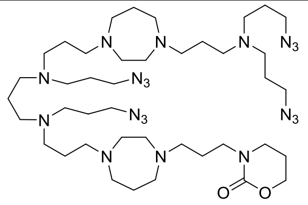
888.6719	$C_{38}H_{77}N_{23}O_2\cdot H^+$	$\boldsymbol{A}_2\boldsymbol{B}\boldsymbol{C}\boldsymbol{D}_5H_3$	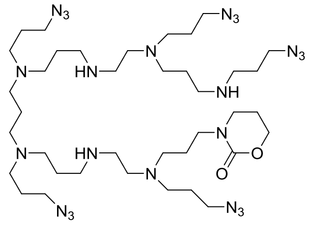
927.7258	$C_{40}H_{82}N_{26}^{+}\cdot H^+$	$\boldsymbol{A}_2\boldsymbol{B}_2\boldsymbol{D}_6H_2$	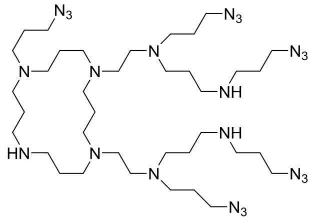
928.7016	$C_{41}H_{81}N_{23}O_2\cdot H^+$	$\boldsymbol{A}_2\boldsymbol{B}_2\boldsymbol{C}\boldsymbol{D}_5H$	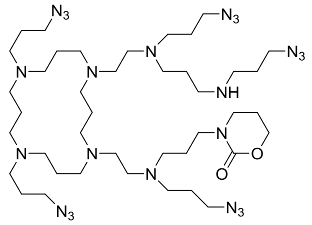
967.7602	$C_{43}H_{86}N_{26}\cdot H^+$	$\boldsymbol{A}_2\boldsymbol{B}_3\boldsymbol{D}_6$	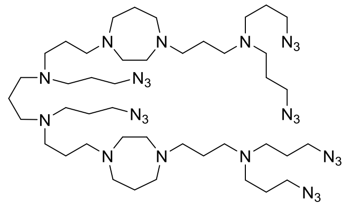
971.7187	$C_{41}H_{82}N_{26}O_2\cdot H^+$	$\boldsymbol{A}_2\boldsymbol{B}\boldsymbol{C}\boldsymbol{D}_6H_2$	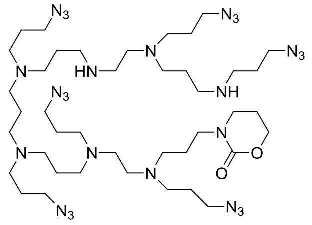

1010.7772	$C_{43}H_{87}N_{29}\cdot H^+$	***A***$_2$***B***$_2$***D***$_7$H	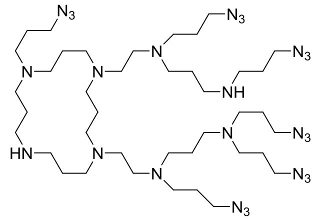
1011.7500	$C_{44}H_{86}N_{26}O_2\cdot H^+$	***A***$_2$***B***$_2$***CD***$_6$	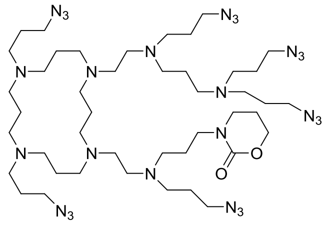
1054.7670	$C_{44}H_{87}N_{29}O_2\cdot H^+$	***A***$_2$***BCD***$_7$H	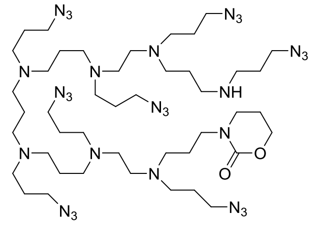
1093.8255	$C_{46}H_{92}N_{32}\cdot H^+$	***A***$_2$***B***$_2$***D***$_8$	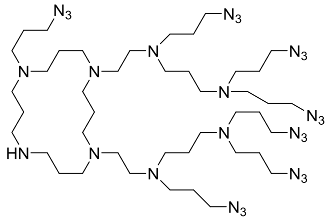
1094.7983	$C_{47}H_{92}N_{29}O_2$	***A***$_2$***B***$_2$***CD***$_7^+$	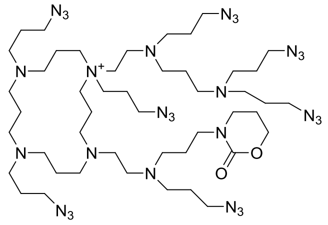
1137.8154	$C_{47}H_{92}N_{32}O_2\cdot H^+$	***A***$_2$***BCD***$_8$	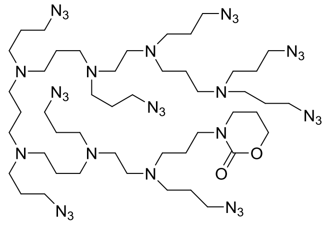

[a]For the sake of clarity, we must outline here that these structures, in particular the ones related to species deriving from the dimerization of the parent amine **4**, are only tentative representations and that in various cases the number of possible isomers is very large (> 20). [b]***A*** = $>N(CH_2)_3\text{-}N(CH_2)_2N\text{-}(CH_2)_3N<$; ***B*** = $\text{-}(CH_2)_3\text{-}$; ***C*** = $\text{-}(CH_2)_3\text{-}O\text{-}CO\text{-}$; ***D*** = $\text{-}(CH_2)_3N_3$

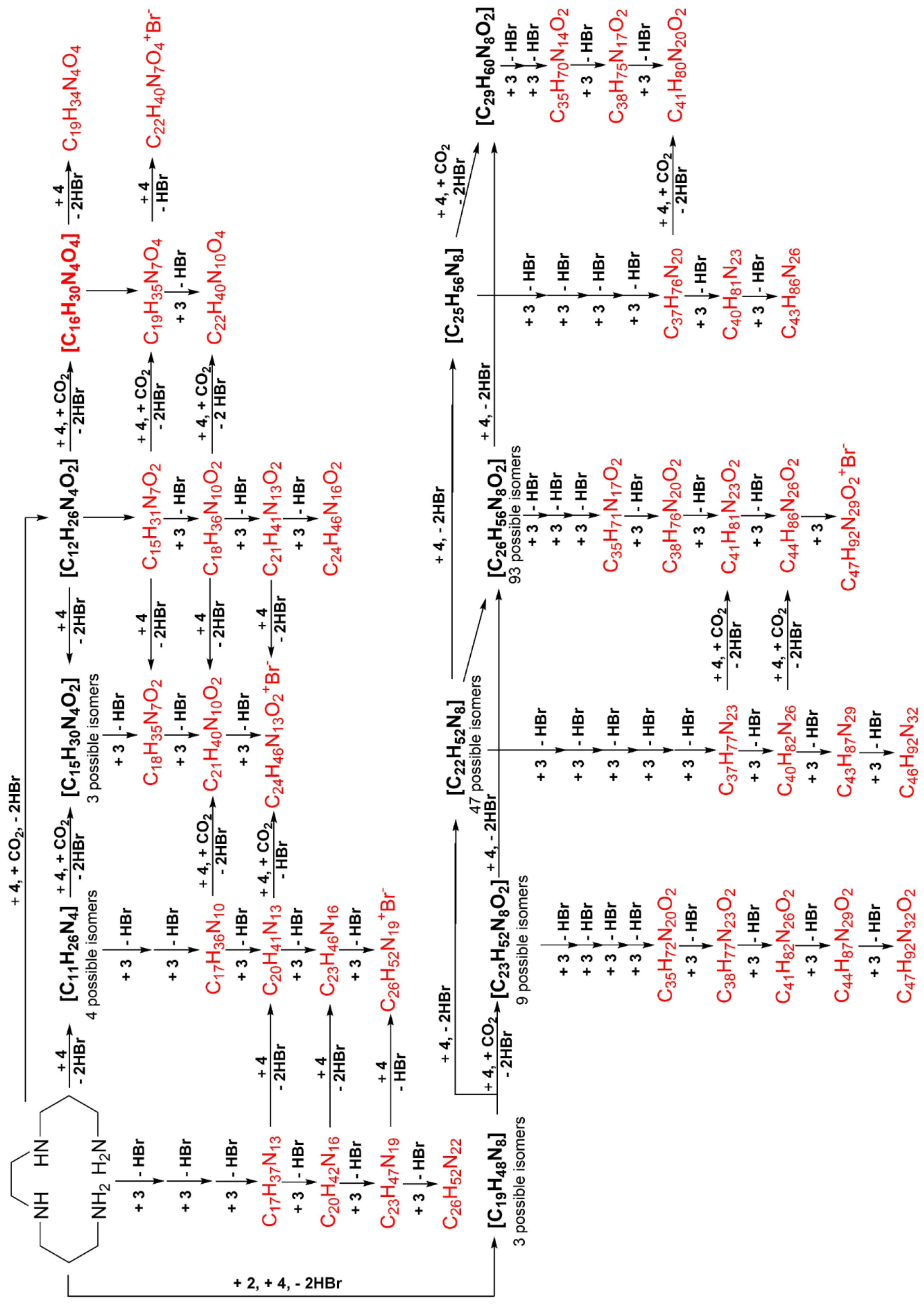

Scheme S1: Formation of the components of mixture **II**.

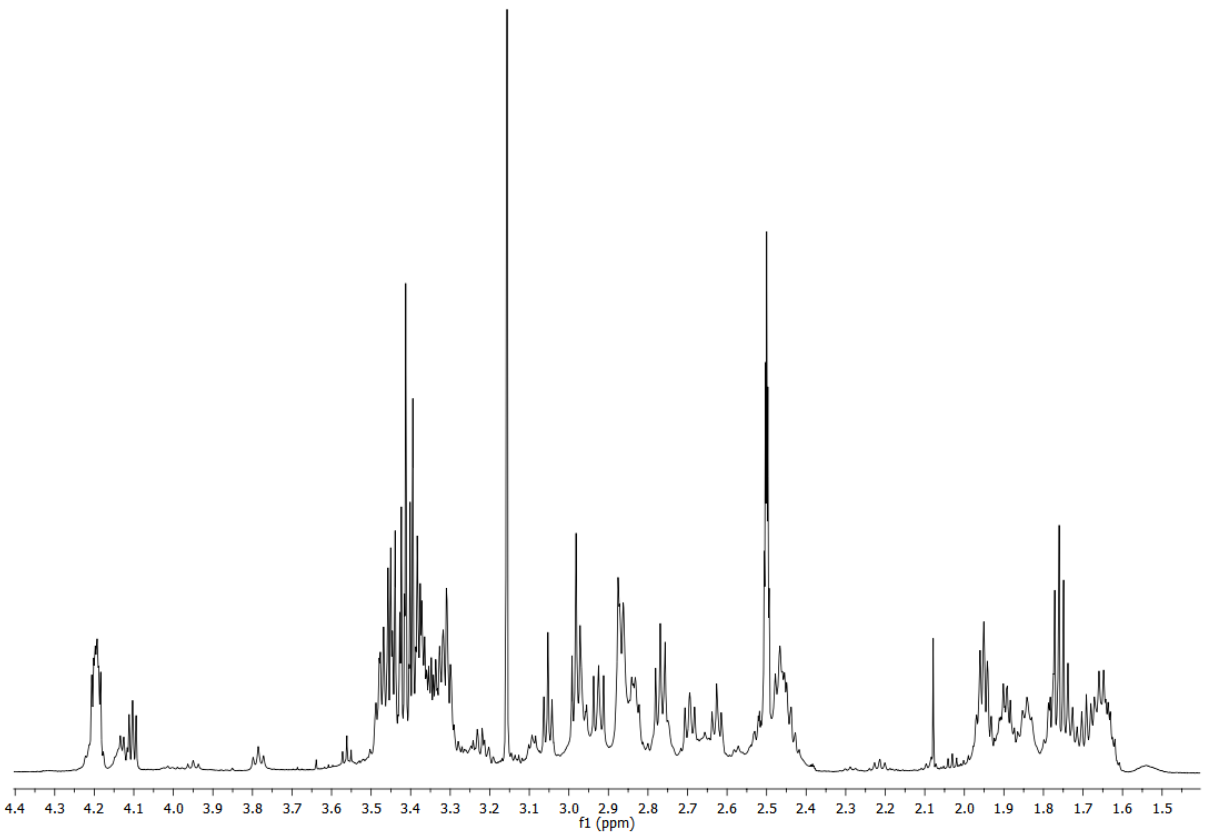

Figure S1: ^{1}H NMR spectrum (DMSO, 600 MHz) of mixture **I**.

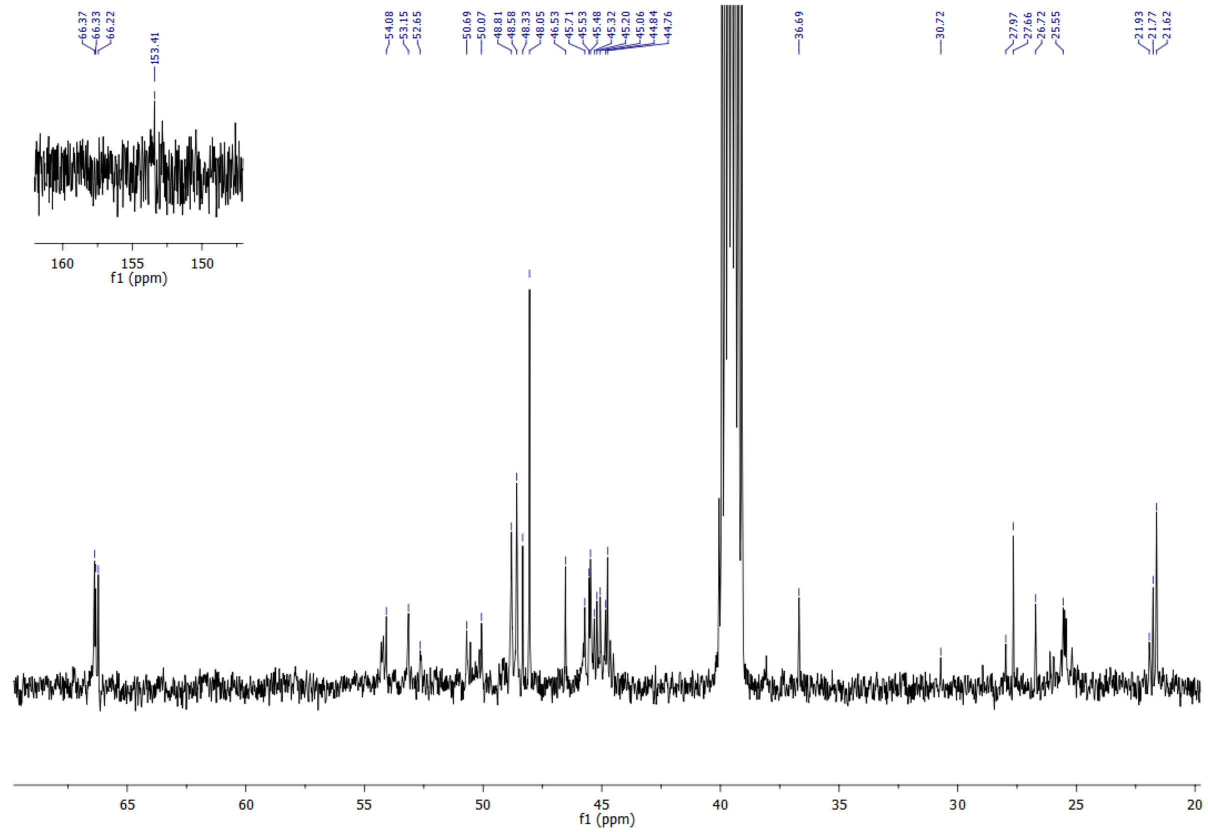

Figure S2: ^{13}C NMR spectrum (DMSO, 150 MHz) of mixture **I**.

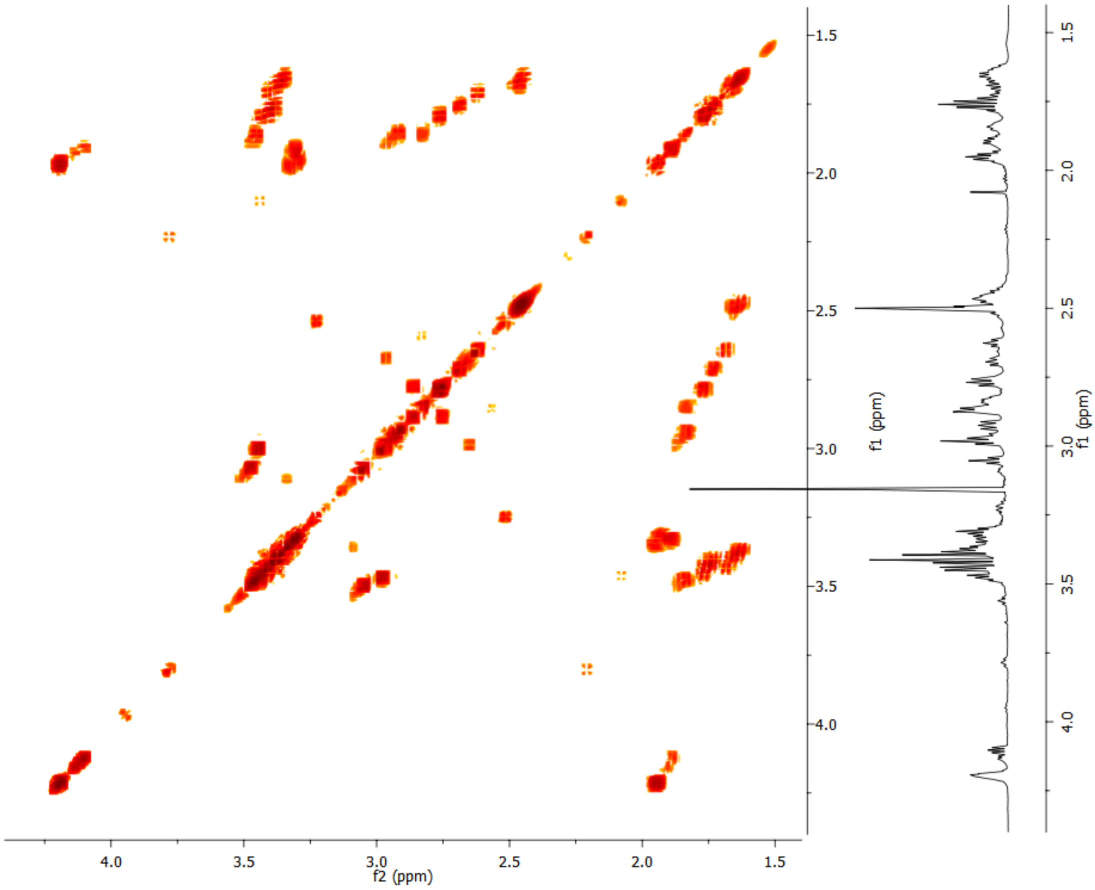

Figure S3: COSY NMR spectrum (DMSO, 600 MHz) of mixture **I**.

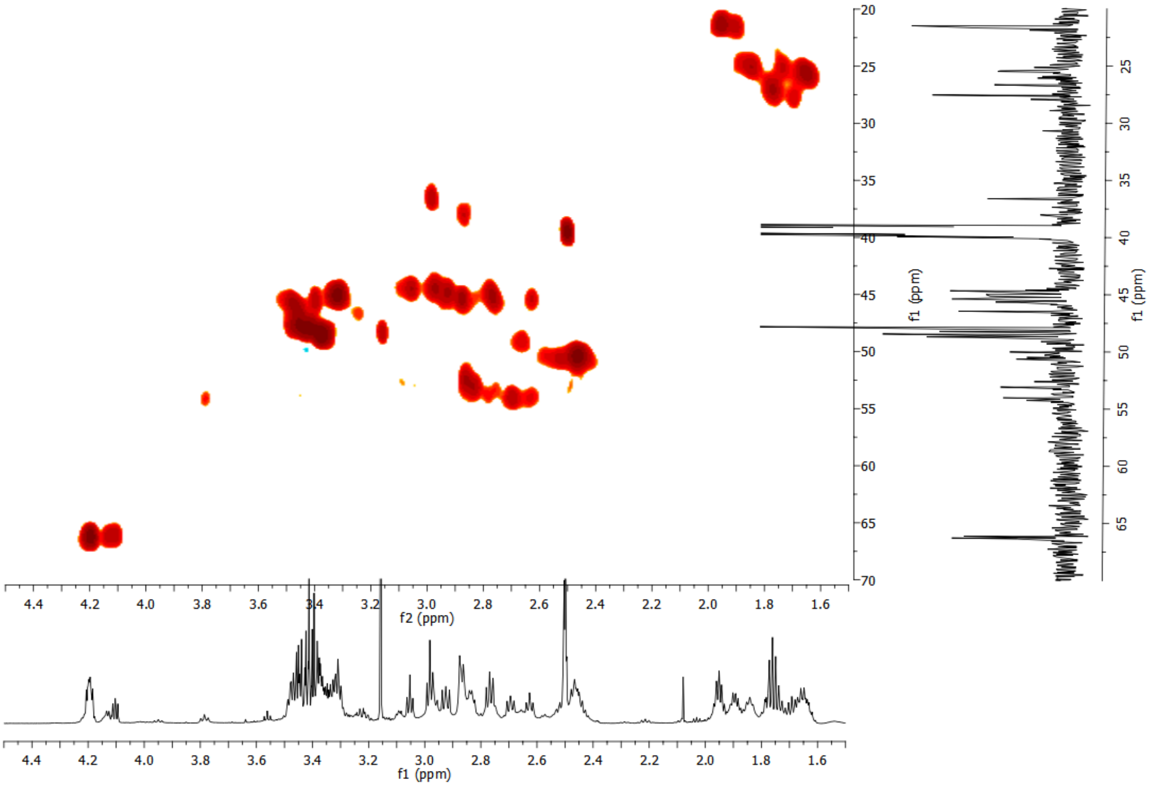

Figure S4: HMQC NMR spectrum (DMSO, 600 MHz) of mixture **I**.

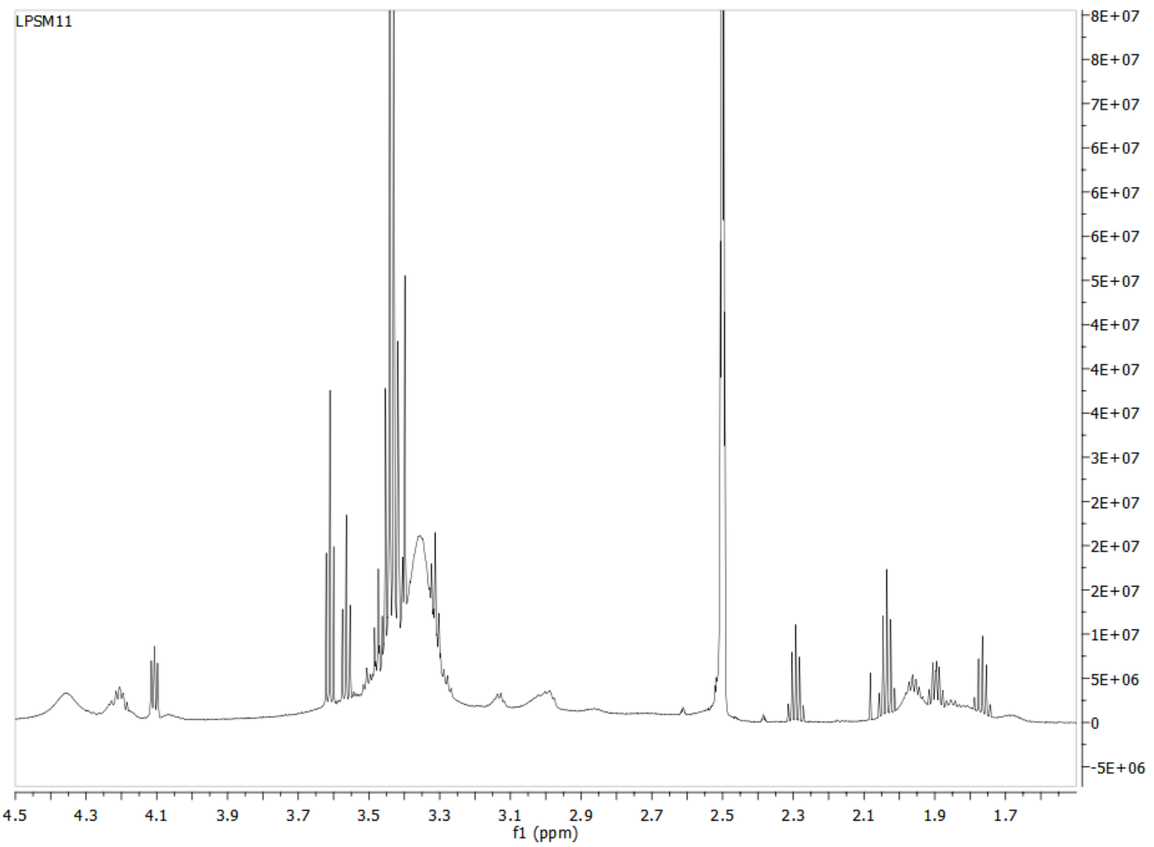

Figure S5: ^{1}H NMR spectrum (DMSO, 600 MHz) of mixture **II**.

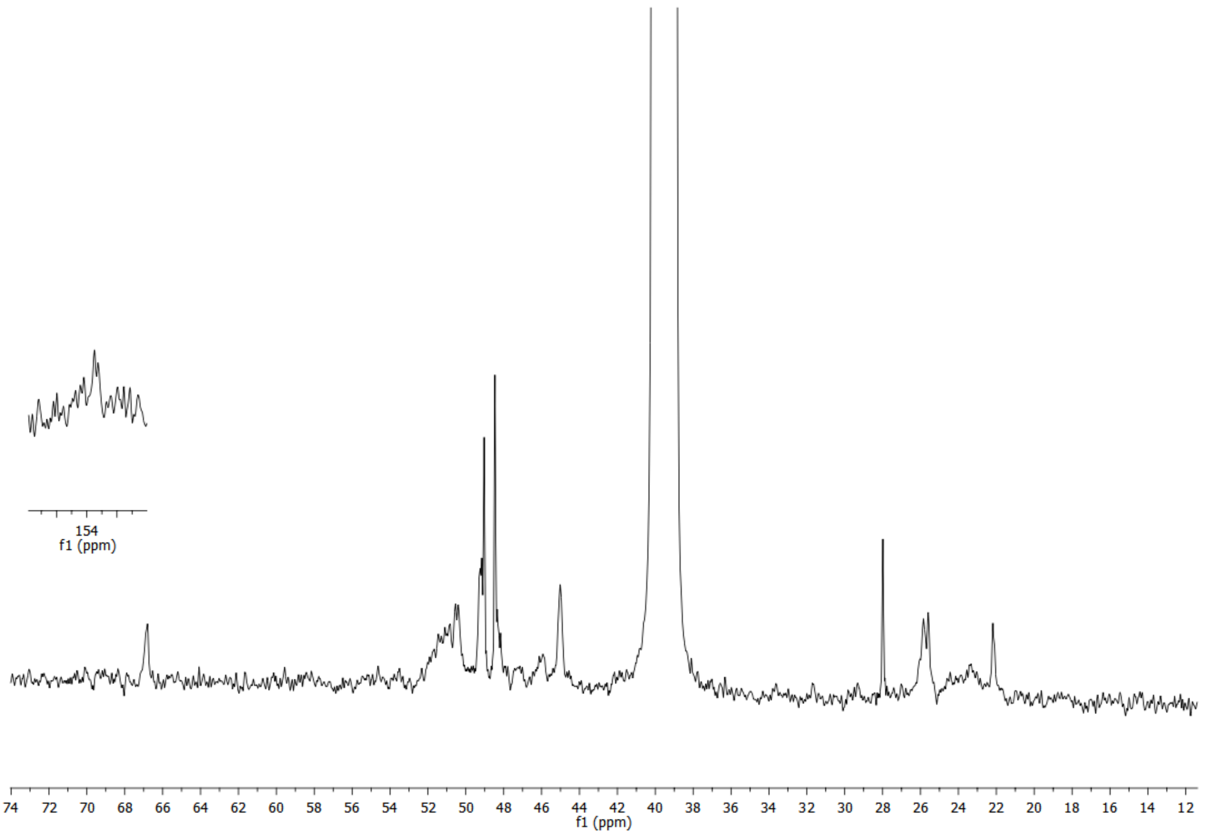

Figure S6: ^{13}C NMR spectrum (DMSO, 150 MHz) of mixture **II**.

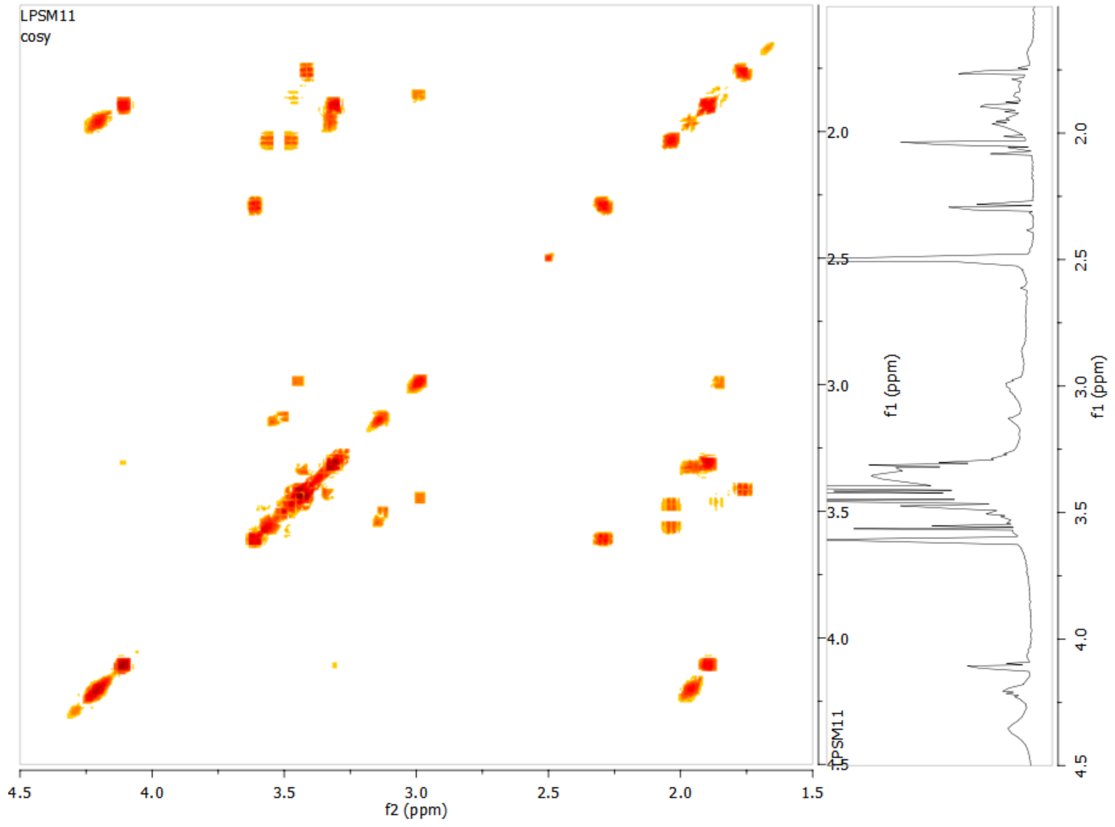

Figure S7: COSY NMR spectrum (DMSO, 600 MHz) of mixture **II**.

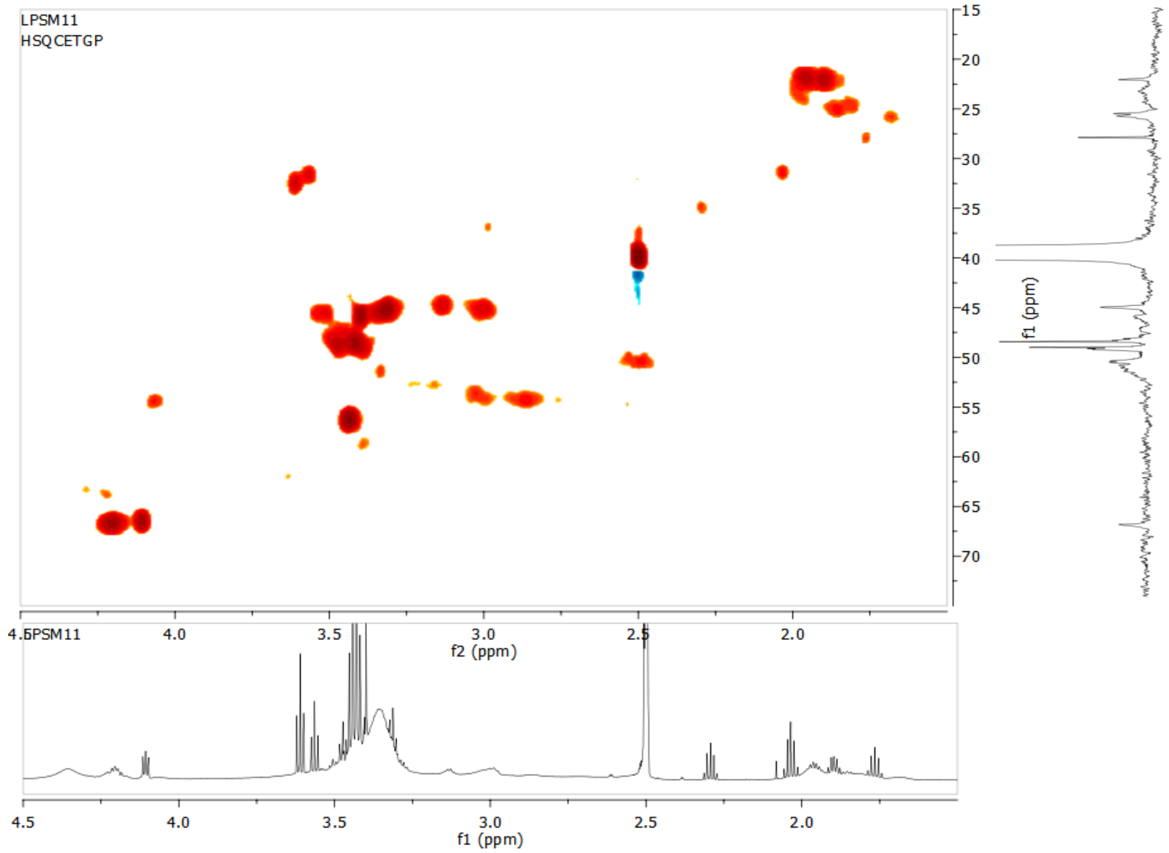

Figure S8: HMQC NMR spectrum (DMSO, 600 MHz) of mixture **II**.

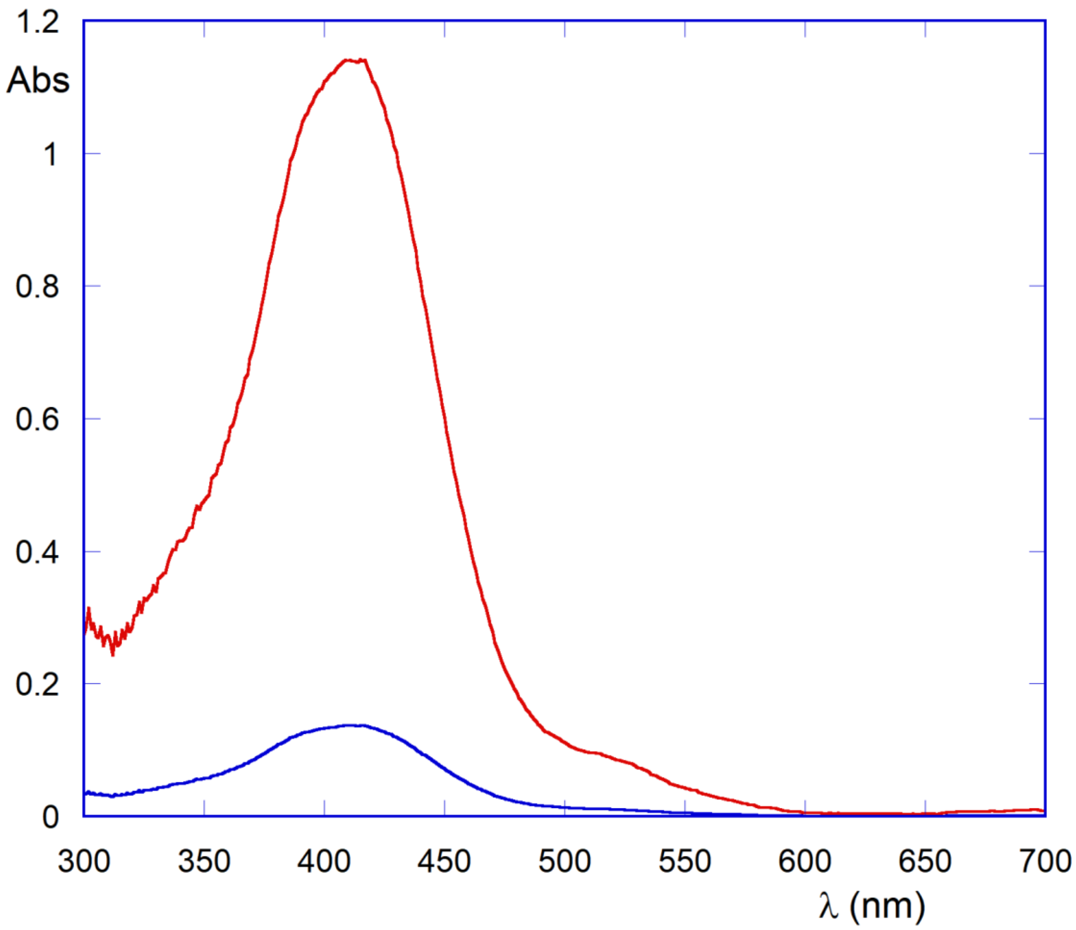

Figure S9: UV–vis spectrum of guest 7 before (red) and after (blue) equilibration with material CaNS-II.
